# Supplementary material for: Intra-condensate demixing of TDP-43 inside stress granules generates pathological aggregates
Source: bioRxiv. 2024 May 24:2024.01.23.576837. Preprint. [Version 3] doi: 10.1101/2024.01.23.576837 (PMC10849624; doi:10.1101/2024.01.23.576837)

bioRxiv preprint doi: https://doi.org/10.1101/2024.01.23.576837; this version posted May 24, 2024. The copyright holder for this preprint (which was not certified by peer review) is the author/funder, who has granted bioRxiv a license to display the preprint in perpetuity. It is made available under aCC-BY 4.0 International license.

SUPPLEMENTAL FIGURE LEGENDS

Figure S1. Intra-condensate demixing and aggregate formation of TDP-43 *in vivo*, related to Figure 1

(A) Fluorescent intensity curve of recombinant GFP-tagged TDP-43 at different concentrations.

(B) Representative images of HeLa cells with low, medium and high expression of TDP-43$^{\Delta NLS}$ before stress during live-cell imaging. High-contrast images are also shown to highlight the low and medium expression cells. The cellular and nucleus boundaries are indicated by dashed lines. Arrow heads indicate small puncta prior to stress in high expression cells. Scale bar, 10 μm.

(C) FRAP of TDP-43$^{\Delta NLS}$ and G3BP1 in stress granules in low expression cells after addition of 100 μM arsenite for 60 min. Data represent the mean ± SD.

(D) FRAP of TDP-43$^{\Delta NLS}$ puncta (G3BP1-negative) in high expression cells before stress and after addition of 100 μM arsenite for 60 min. Data represent the mean ± SD.

(E) Representative confocal images of HeLa cells expressing wild-type TDP-43 with intact NLS after addition of 100 μM arsenite for 120 min. Scale bar, 10 μm and 5 μm for confocal and zoomed in images, respectively.

(F) Representative images of demixed TDP-43$^{\Delta NLS}$ puncta fusion during live-cell imaging. Puncta undergoing fusion are indicated by white arrowheads. Scale bars, 5 μm.

(G) TDP-43$^{\Delta NLS}$ concentrations in HeLa cells in the cytosol before stress (cytosolic), in stress granules at the beginning of demixing (stress granule) and in demixed TDP-43$^{\Delta NLS}$ puncta at the end of demixing (demixed).

(H) Phosphorylation of TDP-43$^{\Delta NLS}$ aggregation upon intra-condensate demixing by confocal microscopy. Cells expressing TDP-43$^{\Delta NLS}$ were stressed with 100 μM arsenite and images were acquired before and after demixing. Scale bar, 10 μm and 5 μm for confocal and zoomed in images, respectively.

(I) Cytoplasmic TDP-43$^{\Delta NLS}$ aggregates generated from intra-condenste demixing recruiting nuclear TDP-43 by confocal microscopy. Cells cotransfected with GFP-tagged TDP-43$^{\Delta NLS}$ (100 ng) and Myc-tagged TDP-43 (50 ng) with intact NLS were stressed with 100 μM arsenite for 120 min. Scale bar, 10 μm and 5 μm for confocal and zoomed in images, respectively.

bioRxiv preprint doi: https://doi.org/10.1101/2024.01.23.576837; this version posted May 24, 2024. The copyright holder for this preprint (which was not certified by peer review) is the author/funder, who has granted bioRxiv a license to display the preprint in perpetuity. It is made available under aCC-BY 4.0 International license.

Figure S2. Intra-condensate demixing and aggregate formation of TDP-43 in reconstituted stress granules, related to Figure 2

(A) Reduced RNA binding by TDP-43 upon intra-condensate demixing in minimal stress granules at 10 h. The TDP-43-rich phase contains less RNA stained by BoBo-3 dye (1 μM) than the G3BP1-rich phase. Scale bar for Figure S2, 10 μm.

(B) Representative condensates in minimal stress granules at 10 h are shown before and after bleaching. The sites of bleaching for mixed and demixed phases are indicated by red solid and white dashed circles, respectively.

(C) The increasing amytracker staining of demixed TDP-43 along intra-condensate demixing process. Amyloid-specific dye amytracker (2.5 μM) was added into minimal stress granules and the ratio of fluorescent intensity between amytracker and TDP-43 was measured to show the increasing stoichiometry. Data represent the mean ± SD.

(D) TDP-43 aggregation in minimal stress granules by SDD-AGE assay. Stress granules containing TDP-43 (10 μM) at 1 h and 10 h were dissolved by 0.3% sarkosyl and analyzed by SDD-AGE containing 0.3% sarkosyl. TDP-43 monomer and oligomer are indicated accordingly.

(E) Prevention of TDP-43 demixing by HSPB1 in minimal stress granules. The experiment was carried out with titrated concentrations of HSPB1 and representative images at 10 h are shown.

(F) TDP-43 recruitment into reconstituted lysate stress granules. Recombinant G3BP1 (20 μM) and lysate from HeLa cells containing ~2 μg/μl cellular proteins were incubated to form reconstituted lysate stress granules. TDP-43 WT or ΔRRM1-2 (0.5 μM) was included as a client of stress granules.

(G) Intra-condensate demixing of TDP-43 in lysate stress granules. TDP-43 (10 μM) was added into lysate stress granules in the presence of 2.5% dextran.

(H) FRAP of TDP-43 in reconstituted lysate stress granules as in (G) over time. The bleaching sites upon recovery are indicated by white dashed circles. Data represent the mean ± SD.

Figure S3. Oxidation is required for intra-condensate demixing of TDP-43, related to Figure 3

(A) Effects of different stressors on intra-condensation demixing of TDP-43$^{\Delta NLS}$ in HeLa cells by confocal microscopy. Cells expressing TDP-43$^{\Delta NLS}$ were

bioRxiv preprint doi: https://doi.org/10.1101/2024.01.23.576837; this version posted May 24, 2024. The copyright holder for this preprint (which was not certified by peer review) is the author/funder, who has granted bioRxiv a license to display the preprint in perpetuity. It is made available under aCC-BY 4.0 International license.

treated with paraquat alone (5 mM) for oxidation, VER-155008 (10 μM) for Hsp70 inhibition or MG132 (10 μM) for proteasome inhibition in the presence of puromycin (10 μg/ml) for 120 min. Scale bar, 10 μm and 5 μm for confocal and zoomed in images, respectively.

(B) TDP-43$^{\Delta NLS}$ concentrations inside stress granules after addition of puromycin (10 μg/ml) in HeLa cells for 180 min.

(C) The threshold concentration for intra-condensate demixing of TDP-43 in minimal stress granules in the absence or presence of GSSG (1 mM).

Figure S4. Homotypic interactions mediated by HP interactions and disulfide bond formation govern TDP-43 demixing, related to Figure 4

(A) One-dimensional contact map showing per-residue interaction probabilities which were computed through the summation of all pairwise interactions for each residue position from the two-dimensional contact map in Figure 4B.

(B) Comparison of per-residue α-helix fraction for CTD of TDP-43 (267–414 aa) between MD simulations in condensed phase and solution NMR. α-helix fractions were computed based on DSSP secondary structure definitions for MD simulations over 25 chains for the 2.5 μs trajectory, and secondary chemical shifts using the delta-2D program for NMR. Data represent the mean ± SEM.

(C) dRMSD of C_α atoms as a function of time with respect to the initial conformation (t=0) for RRM1 (left) and RRM2 (right). Stable RRM domains (dRMSD<0.4 nm) are shown in light gray while unstable domains (dRMSD>0.4 nm) are colored differently.

(D) Comparison between per-residue RMSF for RRM2 domain in the dilute and condensed phase from atomistic MD simulations. The mean RMSF was computed over three independent trajectories (4.5 μs each) for the dilute phase (monomer) and 25 chains (2.5 μs each) for the condensed phase, respectively. Data represent the mean ± SD.

(E) Conformational stability of RRM1 cysteine variants in comparison to the wild-type based on RMSF analysis at 300 K. RMSF for each residue was calculated over three independent trajectories (2.0 μs each, excluding the first 500 ns). Data represent the mean ± SD.

(F) ^{1}H-^{15}N heteronuclear single quantum coherence (HSQC) spectra of wild-type RRM1-2 (102–269 aa, black) and RRM1-2 C173V/C175I (orange) (left). The spectra were recorded at 298 K using 500 μM protein in 50 mM KPi

bioRxiv preprint doi: https://doi.org/10.1101/2024.01.23.576837; this version posted May 24, 2024. The copyright holder for this preprint (which was not certified by peer review) is the author/funder, who has granted bioRxiv a license to display the preprint in perpetuity. It is made available under aCC-BY 4.0 International license.

buffer (pH 6.8) and 150 mM NaCl. Chemical shift assignments were transferred from the BMRB deposited data (BMRB ID: 27613). The chemical shift perturbations of C173V/C175I are mapped on wild-type RRM1-2 (PDB: 4BS2), and 173/175 residues are highlighted in spheres (right). Colors are assigned according to ^{1}H and ^{15}N changes in residues, with ^{1}H >0.05 ppm and ^{15}N >0.1 ppm in magenta, ^{1}H <0.05 ppm and ^{15}N <0.1 ppm in light blue, and residues without substantial change in orange, respectively.

(G) Affinity of TDP-43 variants for A(GU)$_6$ RNA, determined by fluorescence anisotropy. FITC-labeled RNA (5 nM) was added into series diluted TDP-43 in Tris buffer (pH 8.0) containing 150 mM NaCl.

(H) Autoregulation function of TDP-43 in cells. HEK293 cell lines stably expressing HA-tagged wild-type TDP-43 or C173V/C175I upon tetracycline induction for 72 h. Ratio of endogenous TARDBP mRNA and TDP-43 protein between the presence (TC+) and the absence of induction (TC-) are shown, respectively. Data represent the mean ± SD.

(I) Intra-condensate demixing assay for TDP-43 variants. TDP-43 variants (10 µM) were added into the minimal stress granules formed by recombinant G3BP1 (20 µM) and Poly(A) RNA (80 ng/µl) in the presence of 2.5% dextran. Scale bar, 10 µm.

(J) TDP-43 oligomer formation by DLS assay. Wild-type TDP-43 (80 µM) was incubated in 500 mM KCl at 25°C. At the time indicated, aliquots of the samples were diluted into 5 µM in 500 mM KCl and assayed by DLS. Data represent the mean ± SD.

(K) TDP-43 HP$_{mt5}$ maintaining droplet formation. Wild-type TDP-43 or HP$_{mt5}$ (80 µM) was incubated in 500 mM KCl. After 72 h, samples were diluted into a final concentration of 10 µM in 75 mM KCl. Scale bar, 10 µm.

Figure S5. TDP-43 variants with oxidation-resistance and lowered self-assembly propensity abrogate demixing and aggregation *in vivo*, related to Figure 5

(A) Intra-condensate demixing of TDP-43 variants in HeLa cells by STED. Cells expressing TDP-43$^{\Delta NLS}$ variants were stressed with 100 µM arsenite for 120 min and images were taken by STED microscopy. The cellular and nucleus boundaries are indicated by dashed lines. Scale bar, 10 µm and 500 nm for confocal and STED images, respectively.

bioRxiv preprint doi: https://doi.org/10.1101/2024.01.23.576837; this version posted May 24, 2024. The copyright holder for this preprint (which was not certified by peer review) is the author/funder, who has granted bioRxiv a license to display the preprint in perpetuity. It is made available under aCC-BY 4.0 International license.

(B and C) The HP region interactions contributing to TDP-43 aggregation in the demixed phase. Cells expressing TDP-43$^{\Delta NLS}$ variants were stressed with 100 μM arsenite for 60 min, and the ratio of fluorescent intensity between HSPB1 (B) or ubiquitin (C) and demixed TDP-43$^{\Delta NLS}$ was quantified.

(D) Quantification of dissolution of TDP-43$^{\Delta NLS}$ aggregates. HeLa cells expressing TDP-43$^{\Delta NLS}$ variants were stressed with 100 μM arsenite for 60 min following 120 min recovery. The demixed TDP-43$^{\Delta NLS}$ aggregates were monitored in cells capable of dissolving stress granules, and data were presented as the fraction of these cells able to dissolve TDP-43$^{\Delta NLS}$ aggregates. Data represent the mean ± SD.

bioRxiv preprint doi: https://doi.org/10.1101/2024.01.23.576837; this version posted May 24, 2024. The copyright holder for this preprint (which was not certified by peer review) is the author/funder, who has granted bioRxiv a license to display the preprint in perpetuity. It is made available under aCC-BY 4.0 International license.

SUPPLEMENTAL VIDEO LEGENDS

Video S1. Low expression of TDP-43$^{\Delta NLS}$ dispersed inside stress granules in HeLa cells, related to Figure 1

HeLa cells with low expression of TDP-43$^{\Delta NLS}$ were stressed by addition of 100 μM arsenite. TDP-43$^{\Delta NLS}$ (green) and stress granules marked by mCherry-tagged G3BP1 (magenta) were visualized. Scale bar, 10 μm.

Video S2. High expression of TDP-43$^{\Delta NLS}$ forming aggregates independent of stress granules in HeLa cells, related to Figure 1

HeLa cells with high expression of TDP-43$^{\Delta NLS}$ were stressed by addition of 100 μM arsenite. TDP-43$^{\Delta NLS}$ (green) and stress granules marked by mCherry-tagged G3BP1 (magenta) were visualized. Scale bar, 10 μm.

Video S3. Medium expression of TDP-43$^{\Delta NLS}$ undergoing intra-condensate demixing inside stress granules in HeLa cells, related to Figure 1

HeLa cells with medium expression of TDP-43$^{\Delta NLS}$ were stressed by addition of 100 μM arsenite. TDP-43$^{\Delta NLS}$ (green) and stress granules marked by mCherry-tagged G3BP1 (magenta) were visualized. Scale bar, 10 μm.

Video S4. Intra-condensate demixing of TDP-43 inside minimal stress granules, related to Figure 2

TDP-43 (10 μM) was added into minimal stress granules formed by G3BP1 (20 μM) and Poly(A) RNA (80 ng/μl) in the presence of 2.5% dextran. TDP-43 (green) and G3BP1 (magenta) were visualized. Scale bar, 10 μm.

Video S5. Dissolution assay of demixed TDP-43$^{\Delta NLS}$ aggregates in HeLa cells, related to Figure 5

HeLa BAC-G3BP1-mCherry cells with medium expression of TDP-43$^{\Delta NLS}$ were stressed by addition of 100 μM arsenite for 60 min and recovery for 120 min. TDP-43$^{\Delta NLS}$ (green) and stress granules marked by mCherry-tagged G3BP1 (magenta) were visualized. Scale bar, 10 μm.

Video S6. Dissolution assay of TDP-43$^{\Delta NLS}$ C175V/ΔHP inside stress granules in HeLa cells, related to Figure 5

HeLa BAC-G3BP1-mCherry cells with medium expression of TDP-43$^{\Delta NLS}$ C175V/ΔHP were stressed by addition of 100 μM arsenite for 60 min and recovery for 120 min. TDP-43$^{\Delta NLS}$ C175V/ΔHP (green) and stress granules

bioRxiv preprint doi: https://doi.org/10.1101/2024.01.23.576837; this version posted May 24, 2024. The copyright holder for this preprint (which was not certified by peer review) is the author/funder, who has granted bioRxiv a license to display the preprint in perpetuity. It is made available under aCC-BY 4.0 International license.

marked by mCherry-tagged G3BP1 (magenta) were visualized. Scale bar, 10 μm.

Video S7. Intra-condensate demixing of TDP-43$^{\Delta NLS}$ inside stress granules in iPS-MN cells, related to Figure 6

iPS-MN cells cotransfected with TDP-43$^{\Delta NLS}$ and G3BP1 were stressed by addition of 100 μM arsenite. TDP-43$^{\Delta NLS}$ (green) and stress granules marked by mCherry-tagged G3BP1 (magenta) were visualized. Scale bar, 10 μm.

Figure S1. Intra-condensate demixing and aggregate formation of TDP-43 *in vivo*, related to Figure 1

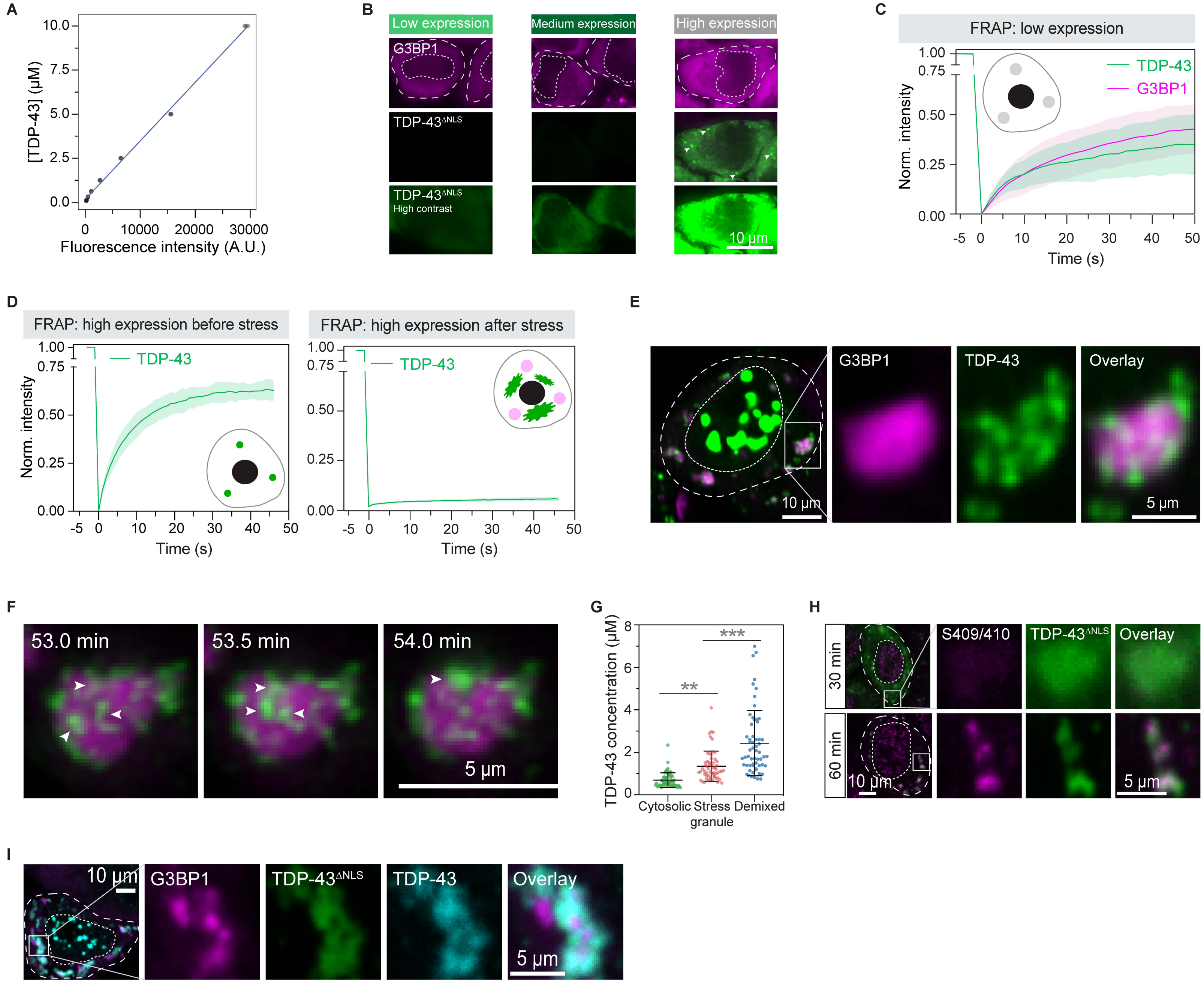

Figure S2. Intra-condensate demixing and aggregate formation of TDP-43 in reconstituted stress granules, related to Figure 2

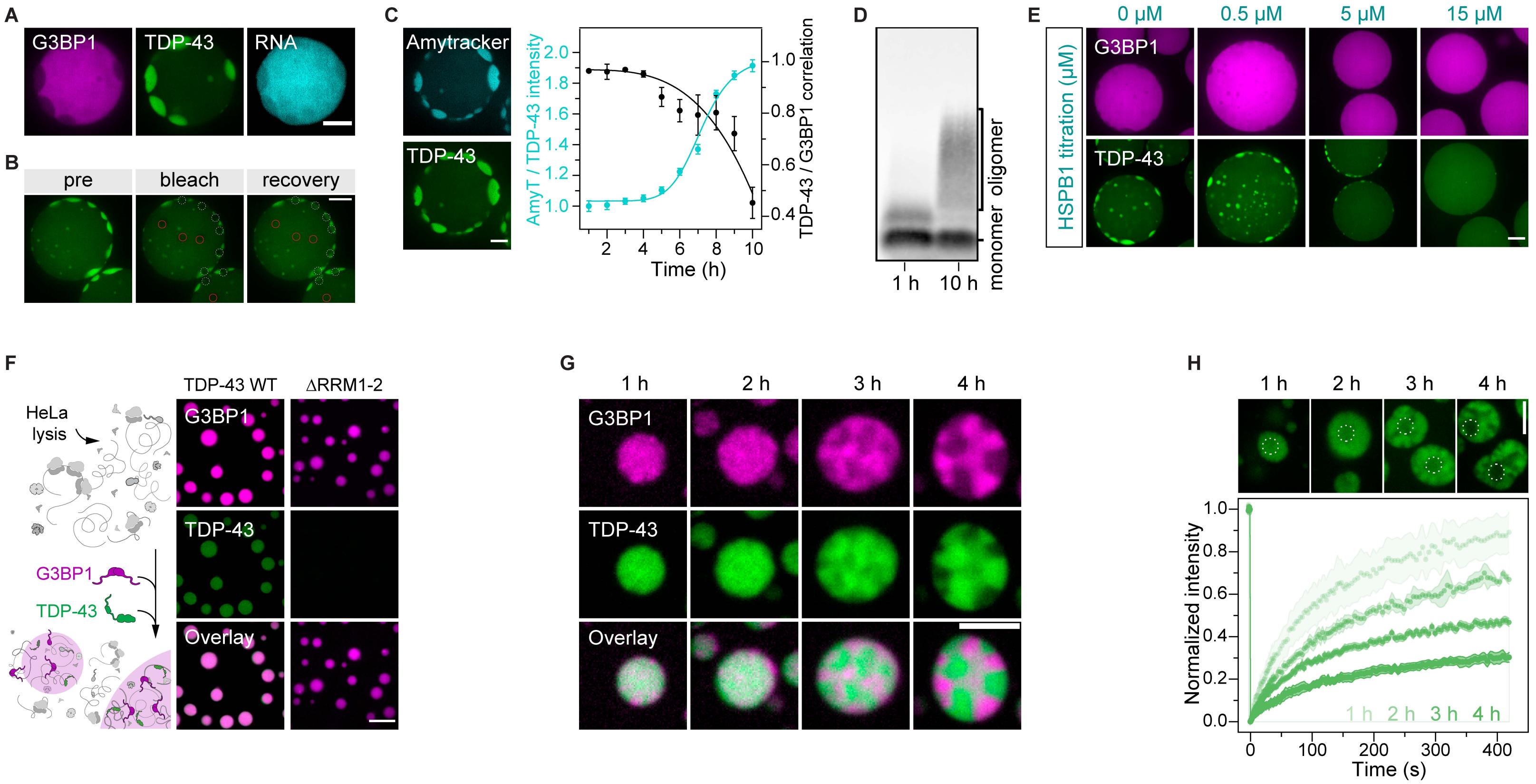

Figure S3. Oxidation is required for intra-condensate demixing of TDP-43, related to Figure 3

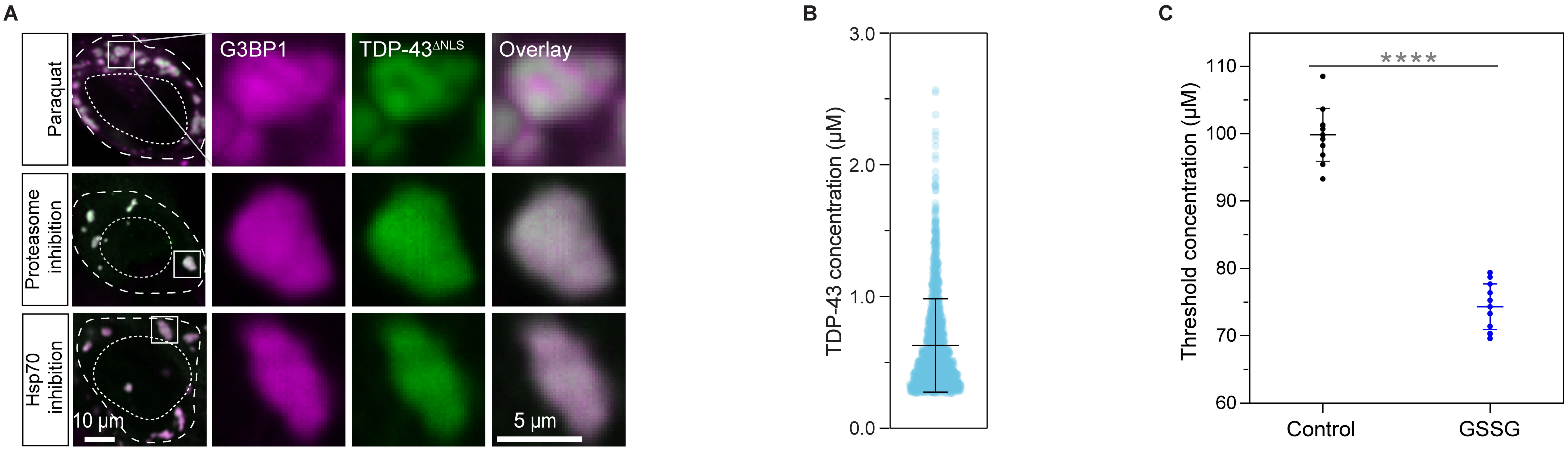

(which was not certified by peer review) is the author/funder, who has granted bioRxiv a license to display the preprint in perpetuity. It is made available under aCC-BY 4.0 International license.

Figure S4. Homotypic interactions mediated by HP interactions and disulfide bond formation govern TDP-43 demixing, related to Figure 4

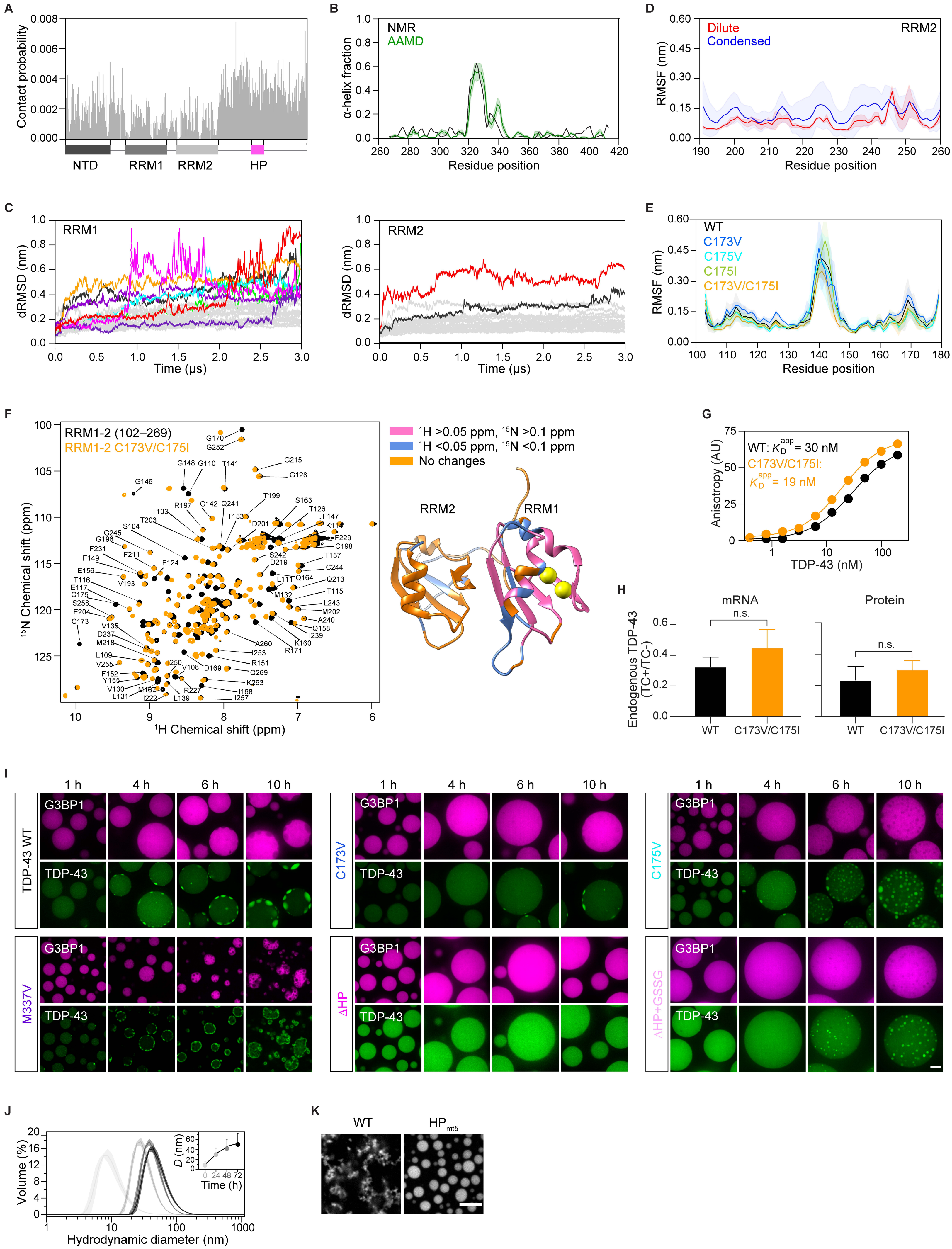

Figure S5. TDP-43 variants with oxidation-resistance and lowered self-assembly propensity abrogate demixing and aggregation *in vivo*, related to Figure 5

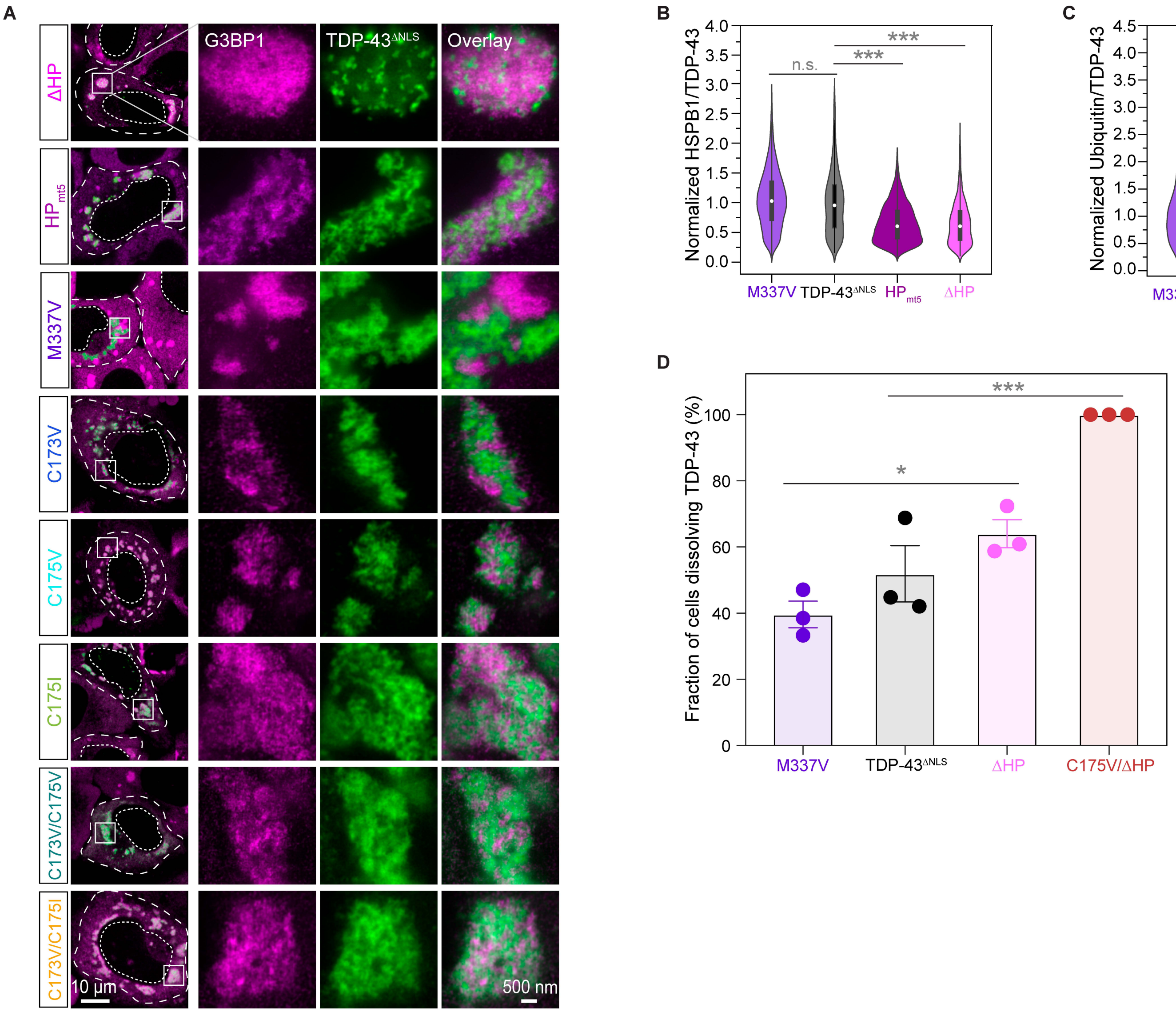

Supplement: Supplement 8 [file NIHPP2024.01.23.576837v3-supplement-8.pdf]
