# Supplementary material for: Intra-condensate demixing of TDP-43 inside stress granules generates pathological aggregates
Source: bioRxiv. 2024 Jan 24:2024.01.23.576837. Preprint. [Version 2] doi: 10.1101/2024.01.23.576837 (PMC10849624; doi:10.1101/2024.01.23.576837)

Figure S1. Intra-condensate demixing and aggregate formation of TDP-43 *in vivo*, related to Figure 1

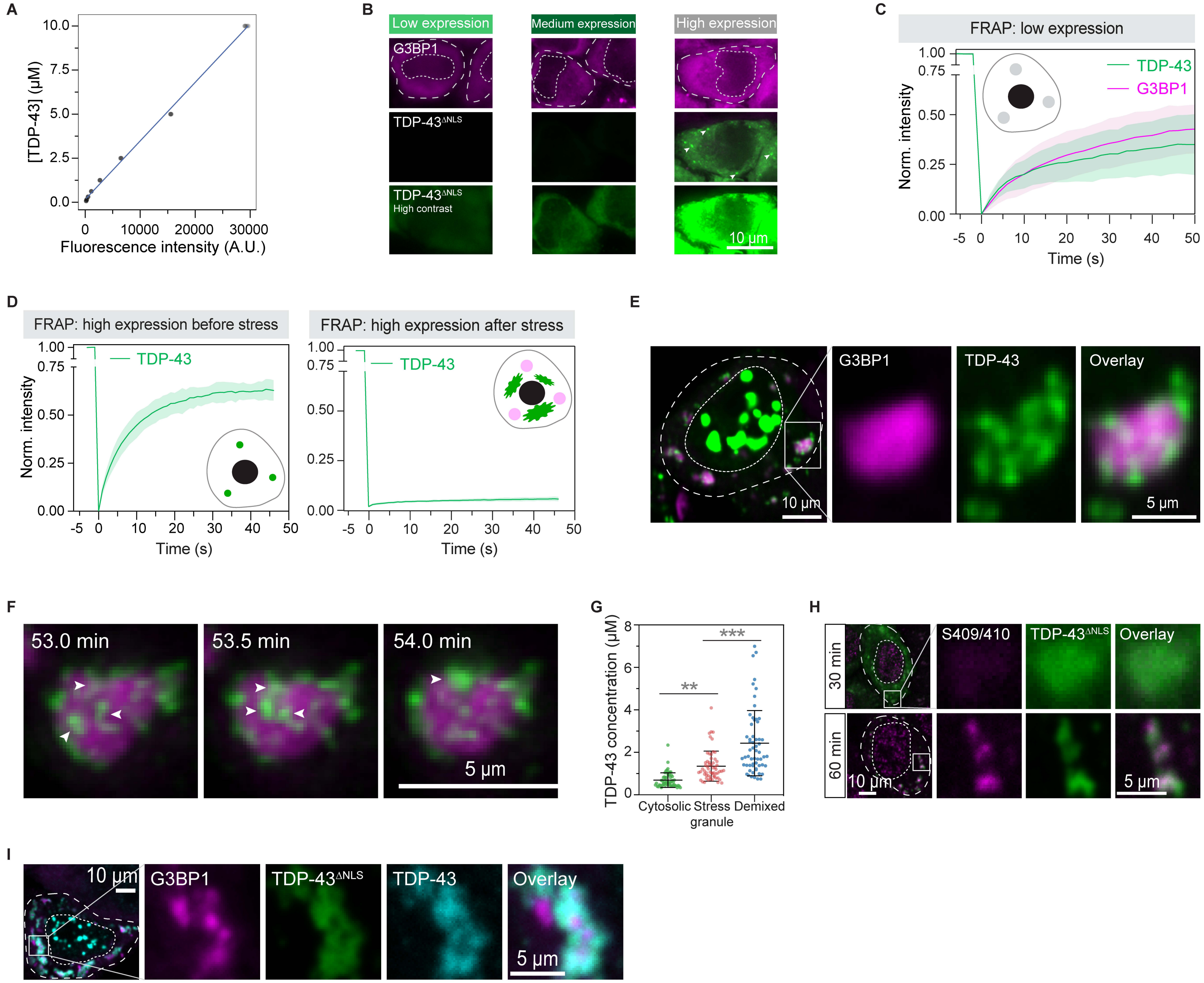

Figure S2. Intra-condensate demixing and aggregate formation of TDP-43 in reconstituted stress granules, related to Figure 2

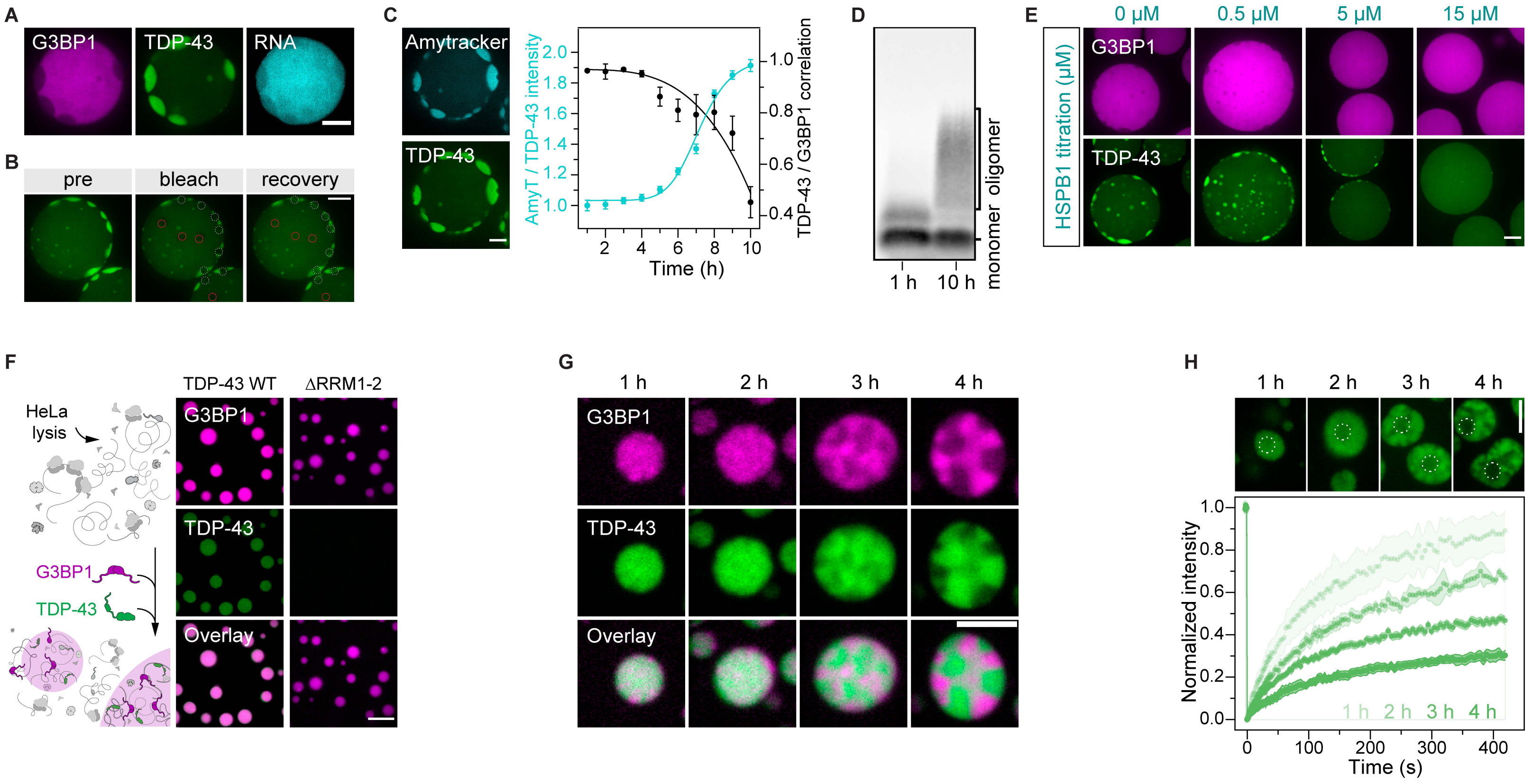

Figure S3. Oxidation is required for intra-condensate demixing of TDP-43, related to Figure 3

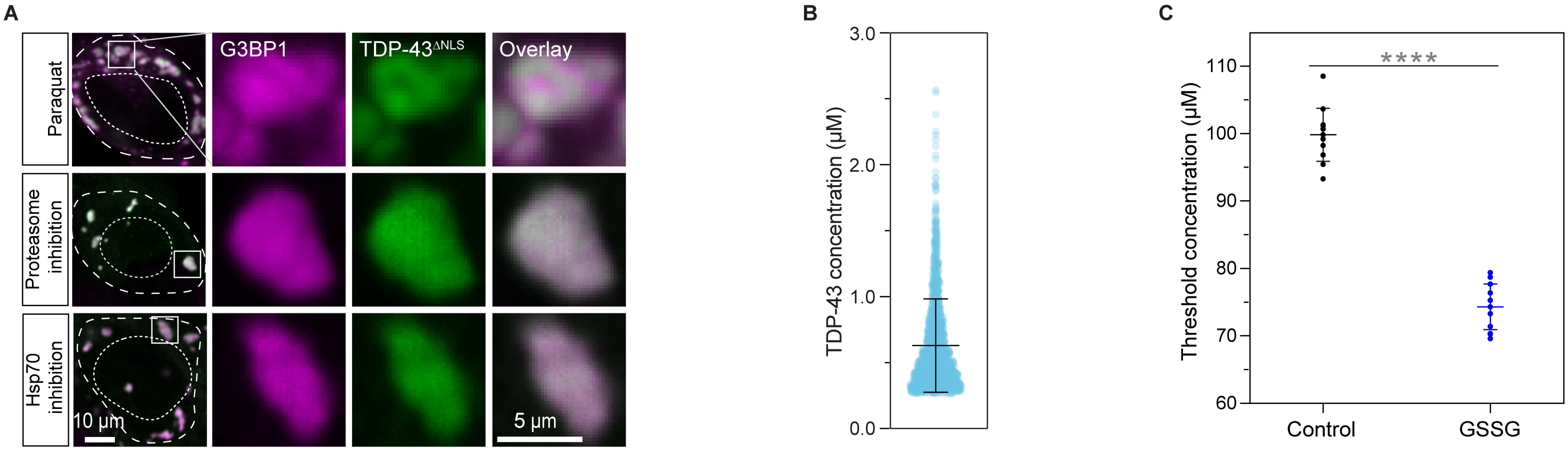

preprint (which was not certified by peer review) is the author/funder, who has granted bioRxiv a license to display the preprint in perpetuity. It is made available under a CC-BY 4.0 International license.

Figure S4. Homotypic interactions mediated by HP interactions and disulfide bond formation govern TDP-43 demixing, related to Figure 4

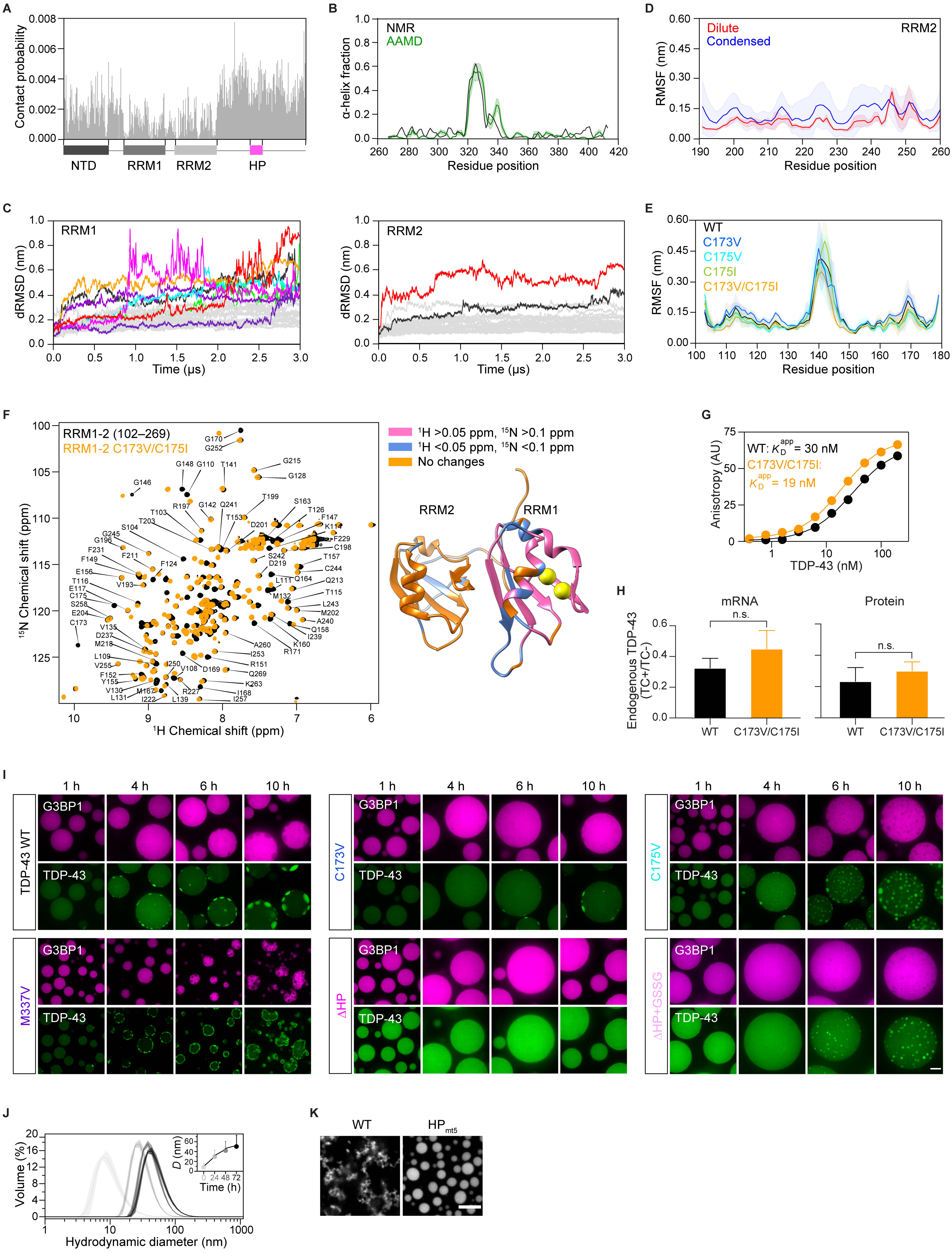

Figure S5. TDP-43 variants with oxidation-resistance and lowered self-assembly propensity abrogate demixing and aggregation *in vivo*, related to Figure 5

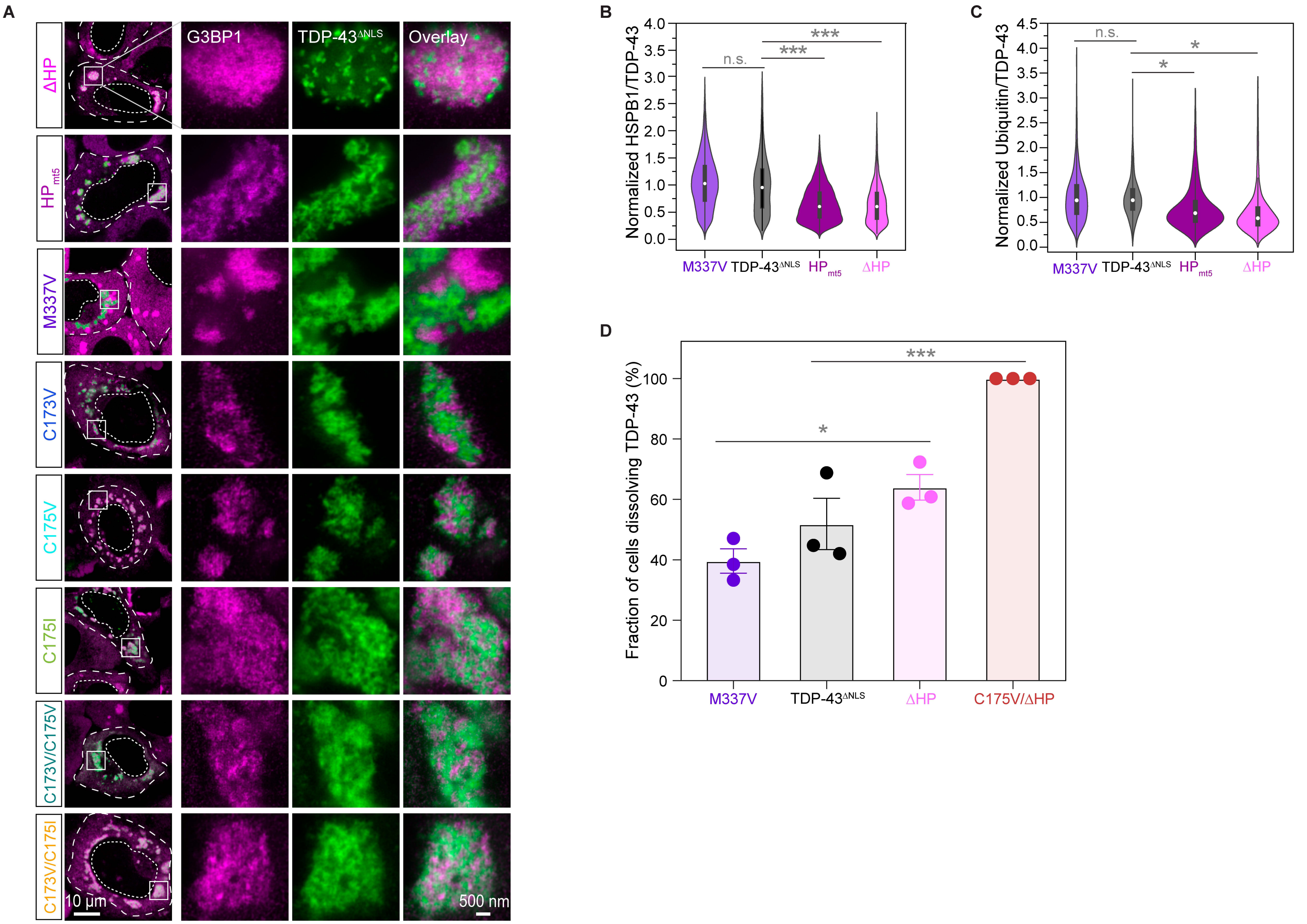

Supplement: Supplementary file 1 [file NIHPP2024.01.23.576837v2-supplement-8.pdf]
